# Systemic Treatment of Orthodontic Elastic Band‐Induced Periodontitis: A Case Report

**DOI:** 10.1002/ccr3.72997

**Published:** 2026-06-29

**Authors:** Limin Liu, Lin Chen, Haijing Gu, Xiayi Wu, Jieyi Chen, Yongbiao Huo

**Affiliations:** ^1^ Hospital of Stomatology, Guanghua School of Stomatology Sun Yat‐Sen University Guangzhou Guangdong China; ^2^ Department of Orthodontics, the Third Affiliated Hospital Sun Yat‐Sen University Guangzhou Guangdong China

**Keywords:** multidisciplinary treatment, oral surgery, orthodontics, periodontics, restorative dentistry

## Abstract

Multidisciplinary Treatment (MDT) approach for immature permanent teeth with severe periodontitis due to an iatrogenic orthodontic elastic band.

## Key Clinical Message

Unsupervised orthodontic elastics for midline diastema closure induce severe periodontal damage. MDT provides comprehensive intervention (including elastic removal, splinting, antibiotics, and orthodontic adjustments) to preserve affected teeth and restore aesthetics, reminding dentists and parents to avoid inappropriate use and emphasize early monitoring for preventing irreversible tooth loss.

## 1 Introduction

Orthodontic elastic bands are widely utilized during orthodontic therapy to assist with space closure, tooth positioning, and anchorage management [1]. However, incorrect placement may cause significant iatrogenic periodontal harm. When the elastic band shifts apically beneath the gingival margin, it functions as a continuous foreign body, aggravating acute inflammatory reactions, disruption of the periodontal ligament, and rapid resorption of alveolar bone [2]. In severe instances, this can result in pathological tooth mobility, extrusion, and even early tooth loss.

Since the 1870s, cases of elastic band‐induced periodontitis and corresponding treatment strategies have been reported in the literature. Diagnosing this atypical and aggressive form of periodontitis is particularly challenging because orthodontic elastics are radiolucent, making them invisible on radiographs and difficult to detect through probing during clinical examination. Additionally, patients often fail to recognize the connection between elastic band use and subsequent periodontal destruction, leading to delayed diagnosis. Although several case reports have documented similar injuries, effective management protocols for cases involving substantial bone loss and immature apices remain scarce [3, 4]. Furthermore, approaches to address esthetic concerns resulting from tooth loss are insufficient. This case report presents a severe periodontal injury affecting young permanent incisors caused by a subgingival displaced orthodontic elastic band. A comprehensive multidisciplinary treatment (MDT) strategy was employed to resolve inflammation, eliminate the foreign body, stabilize the teeth, promote bone regeneration, and restore both occlusion and aesthetics through staged interventions. The objective of this report is to describe a MDT procedure for managing severe periodontitis in immature permanent teeth resulting from improper orthodontic elastic band application, along with subsequent treatment to enhance esthetic outcomes.

## 2 Case History/Examination

**FIGURE 1:**
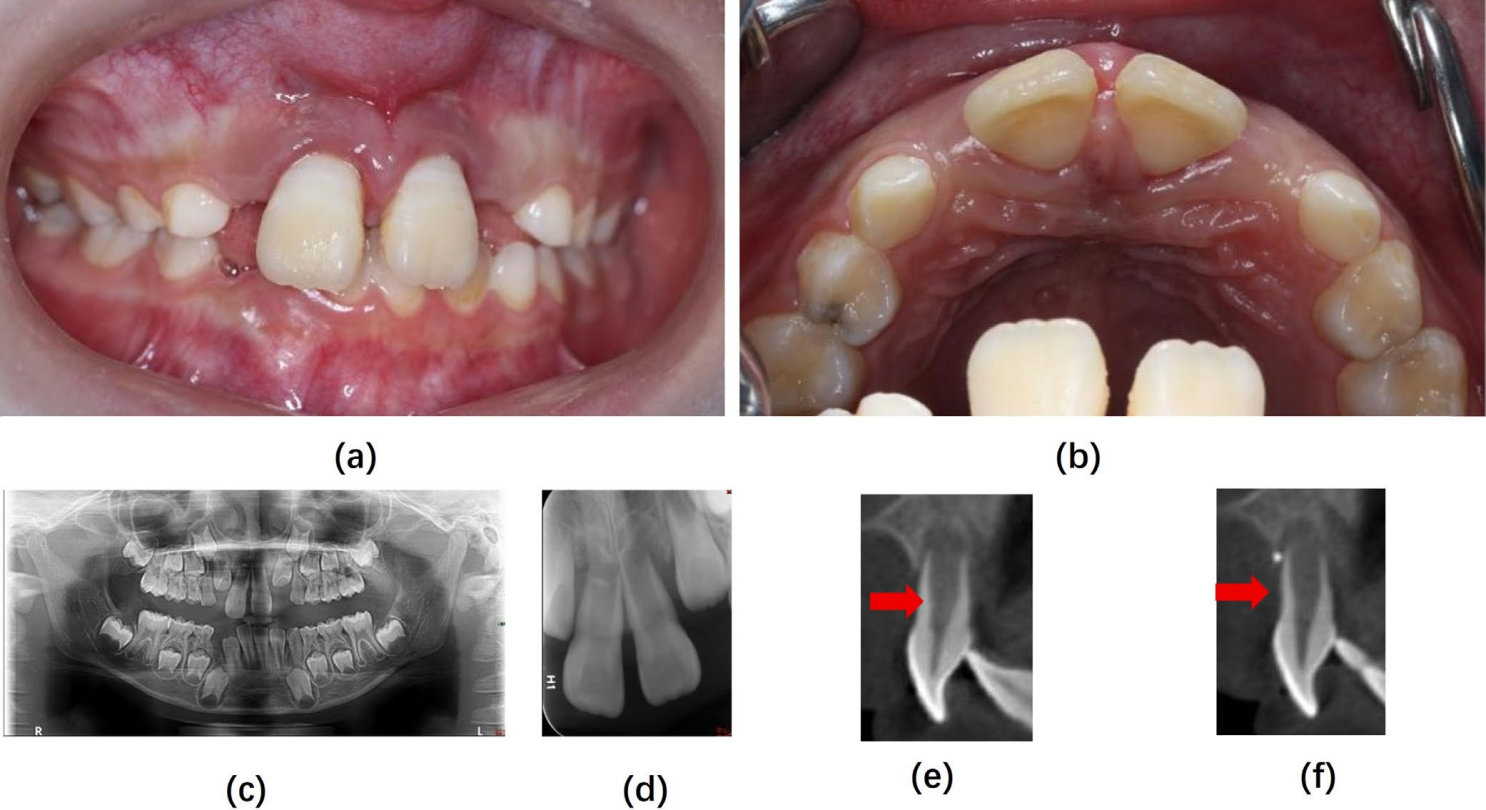
Pre‐surgical intraoral and radiograph views. (a) Labial view showed swelling and misalignment of teeth 11 and 21; (b) occlusion view showed swelling around teeth #11 and #21; (c–f) pre‐surgical radiograph showed mixed dentition, with high‐density shadows on the labial aspect of 11, teeth #11 and #21 and an arch‐like bone defect around the root.

Written informed consent was obtained from the patient for publication of this report in compliance with the journal's consent policy. An 8‐year‐old male patient was referred to the Department of Orthodontics. He presented with mobility and elongation of the maxillary anterior teeth that had been noticed for several weeks. Clinical assessment revealed that both the right maxillary central incisor (#11) and left maxillary central incisor (#21) exhibited elongated clinical crowns and a deep overbite. The gingival tissue surrounding #11 and #21 was dark red with papillary edema. The Sulcus Bleeding Index (SBI) for both teeth was scored at 4, and the Clinical Attachment Level (CAL) measured 8 mm. Both #11 and #21 demonstrated Grade 3 mobility with a probing depth (PD) ranging from 5 to 7 mm. A 2‐mm diastema was noted between #11 and #21. Periapical radiography revealed bone destruction extending apically, presenting as a funnel‐shaped or crater‐like radiolucent area encompassing the apices of both teeth. Panoramic radiography showed no evident bone resorption in other teeth. Cone‐beam computed tomography (CBCT) demonstrated irregular radiolucent lesions in the apical regions of #11 and #21, with small high‐density deposits noted adjacent to the apex of #11. The labial and palatal bone plates of both teeth had been resorbed to approximately half the root length (Figure 1).

## 3 Differential Diagnosis

### 3.1 Diagnosis: Acute Localized Periodontitis (Staging III, Grading C) and Partial Extrusive Dislocation

Diagnostic criteria for Acute localized periodontitis. The diagnosis was based on several key clinical findings. First, we observed localized severe gingival inflammation, characterized by erythema, edema, and spontaneous bleeding surrounding teeth #11 and #21. Clinically, the patient exhibited rapid periodontal deterioration, evidenced by a Sulcus Bleeding Index (SBI) of 4, Clinical Attachment Loss (CAL) of 8 mm, and Probing Depth (PD) ranging from 5 to 7 mm over a few weeks. Additionally, the affected teeth presented Grade III mobility accompanied by pathological extrusion. Radiographically, an arc‐shaped vertical bone loss extending to the periapical area was noted, with almost total loss of the labial and palatal bone plates. Crucially, the etiology was identified as a foreign body reaction; the patient admitted to using an elastic band for diastema closure, and subgingival migration was surgically confirmed. Other causes such as trauma, caries, or pulpitis were ruled out.

### 3.2 Diagnostic Criteria for Partial Extrusive Dislocation

The diagnosis of partial extrusive dislocation was supported by the crown displacement and apparent elongation of teeth #11 and #21. While mobility was significantly increased, the teeth remained in the alveolar socket without avulsion. Furthermore, clinical and radiographic examinations exclude root or alveolar fractures. The radiographic appearance of a widened periodontal ligament space, combined with apical bone loss, was consistent with an extrusive injury pattern.

### 3.3 Differential Diagnosis Is Acute Apical Periodontitis and Orthodontic Root Resorption

(1) Acute apical periodontitis typically presents with spontaneous pain, severe occlusal pain, and non‐vital pulp, usually secondary to caries or pulpitis, without a history of acute displacement. (2) Orthodontic root resorption is characterized by chronic, gradual mobility and radiographic root shortening, without acute traumatic displacement. (3) Generalized aggressive periodontitis generally affects multiple teeth, shows a familial pattern, and involves generalized bone loss; this was excluded because the destruction was confined strictly to #11 and #21, with no other teeth involved (confirmed by panoramic radiography) and no family history of periodontitis.

## 4 Multidisciplinary Treatment

Following a comprehensive multidisciplinary evaluation involving a periodontist, pediatric dentist, oral surgeon, and orthodontist, two distinct therapeutic strategies were formulated: (1) extraction followed by placement of an interim removable prosthesis, with definitive implant rehabilitation deferred until skeletal maturity; or (2) retention of the compromised dentition through coordinated multidisciplinary therapy aimed at restoring both function and aesthetics. After thorough counseling with the parents regarding risks, benefits, and long‐term implications, the second approach—prioritizing tooth preservation—was ultimately chosen.

### 4.1 Preserve the Tooth and Periodontal Surgery

**FIGURE 2:**
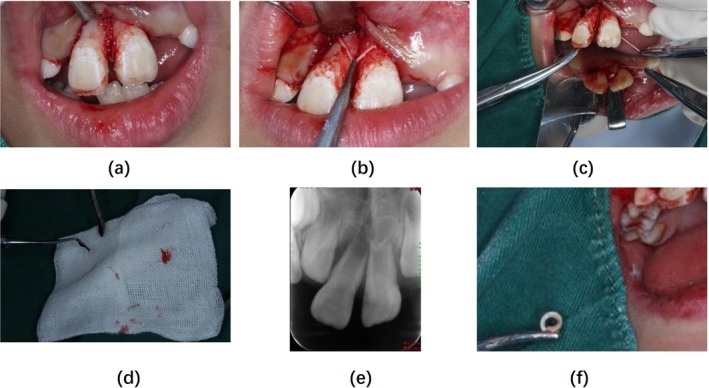
Clinical views during the flap surgery and postoperative radiograph. (a) Surgical site exposed after flap reflection; (b, c) elastic band visualized following flap elevation; (d) resin‐like fragments identified during surgery; (e) removal of the elastic band from the subgingival region; (f) postoperative radiograph showed no residual foreign material.

**FIGURE 3:**
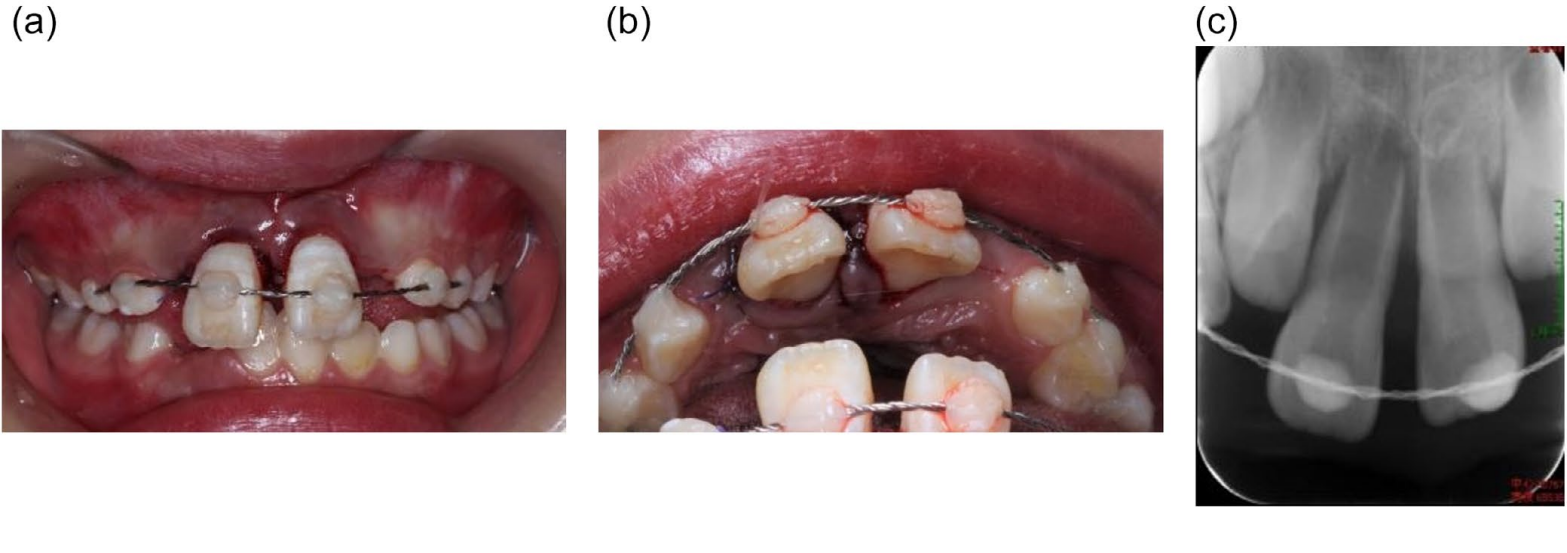
Post‐surgical intraoral and radiograph views of resin splinting. (a) Labial view showed resin splint; (b) occlusal view showed resin splint treatment with resin splint; (c) radiograph confirming resin splint placement.

Under local anesthesia, full‐thickness mucoperiosteal flaps were raised from #12 to #22 by the oral surgeons. Severe labial bone resorption with extensive root exposure was observed. Resin‐like debris and a continuous elastic band encircling the apical third of both #11 and #21 were identified and completely excised. The surgical area was thoroughly irrigated with saline. The elongated incisors were carefully repositioned, and the flaps were closed using non‐absorbable silk sutures (Figure 2). The pediatric dentists stabilized #11 and #21 by splinting them to adjacent teeth with two 16 in. arch wires. Postoperative medications included amoxicillin 500 mg every 8 h for 3 days, ibuprofen 200 mg as needed for pain, and 0.12% chlorhexidine gluconate mouth rinse (10 mL, three times daily). A soft diet and strict oral hygiene instructions were provided. After 18 days of splinting, the gingival appearance had normalized. SBI remained at 4, CAL was 8 mm, and PD had reduced to 5 mm. Thermal testing showed a responsive and comparable result to contralateral teeth. The splint was then removed, and the teeth were polished (Figure 3).

### 4.2 Orthodontic Intrusion and Functional‐Esthetic Rehabilitation

After 12 months of monitoring, low‐force orthodontic treatment was initiated. At this stage, SBI = 2, CAL = 6 mm, PD = 3 mm.

#### 4.2.1 Orthodontic Biomechanics

**FIGURE 4:**
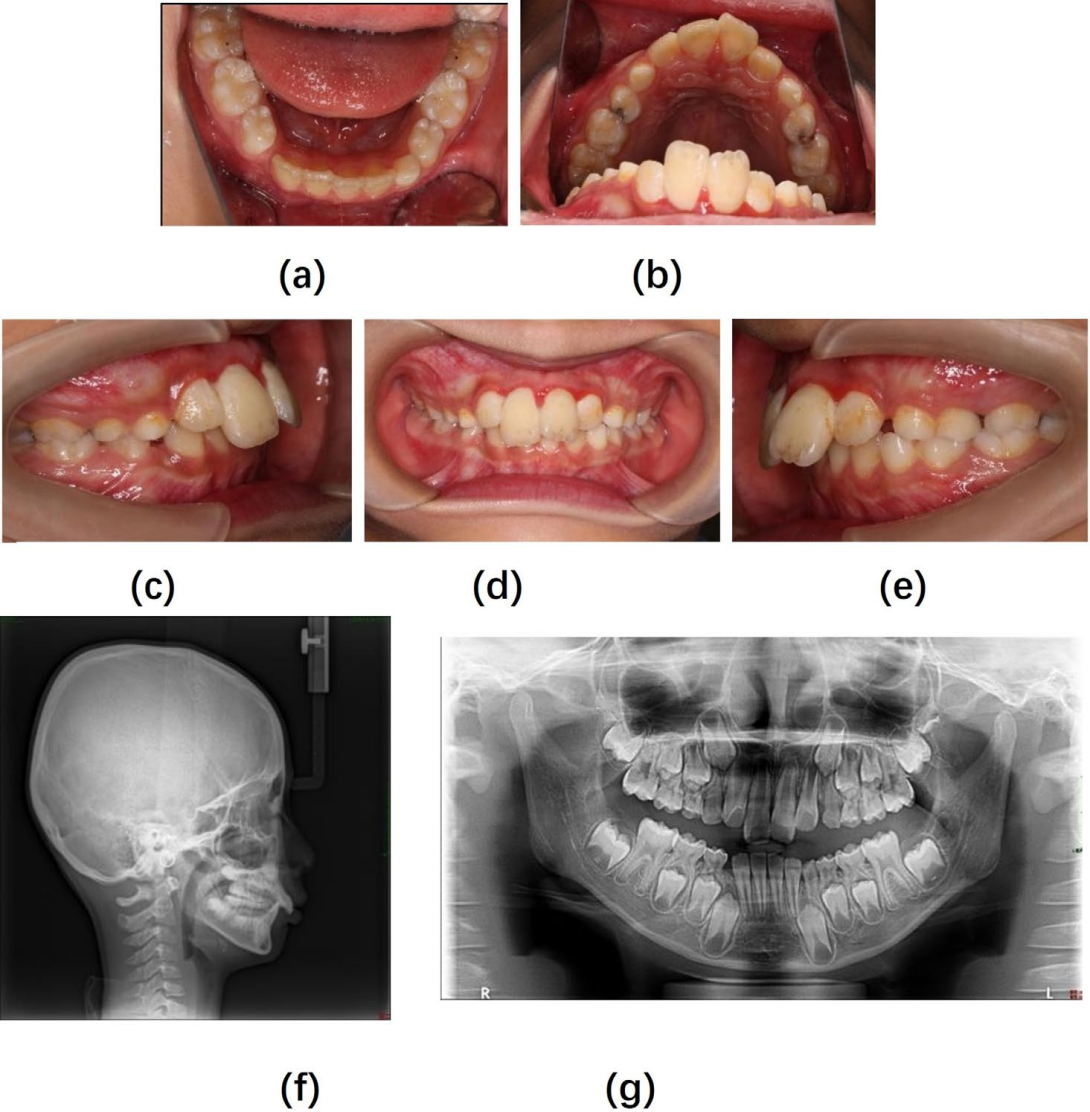
Clinical and radiograph view 12 months after surgery. (a–e) Intraoral view 12 months; (f–g) radiograph view 12 months.

**FIGURE 5:**
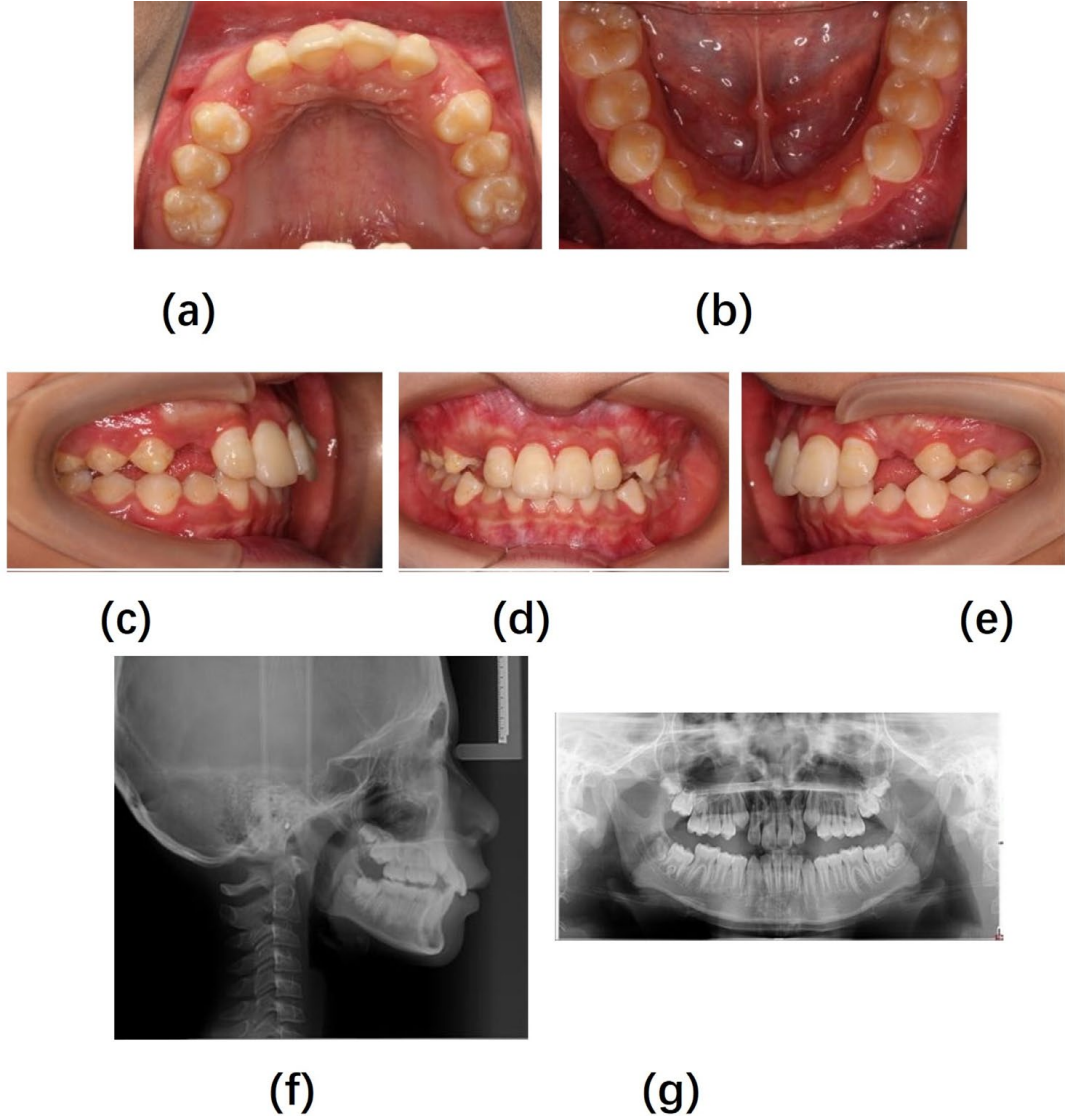
Clinical and radiograph views during orthodontic treatment. (a–e) Intraoral views during orthodontic treatment; (f–g) radiograph views during orthodontic treatment.

A light, continuous intrusive force was applied to minimize stress on the periodontium. The intrusion mechanics were designed to gradually reposition the roots and stimulate alveolar bone regeneration. The force magnitude was reduced by 50% (0.125 mm per stage) to ensure biologically compatible tooth movement. Invisalign clear aligners were selected to deliver controlled, light, intermittent forces while facilitating oral hygiene maintenance. The treatment plan encompassed: (1) lingual torque application to enhance root stability; (2) intrusion of the over‐erupted central incisors; (3) maxillary arch expansion to correct transverse deficiency; and (4) sequential leveling and alignment. Throughout the treatment, plaque control and periodontal maintenance were performed at every visit. Upon completion of active alignment, #11 and #21 demonstrated SBI = 2, CAL = 3–4 mm, PD = 2 mm, with no residual pockets or abnormal mobility (Figures 4 and 5).

## 5 Treatment Results

**FIGURE 6:**
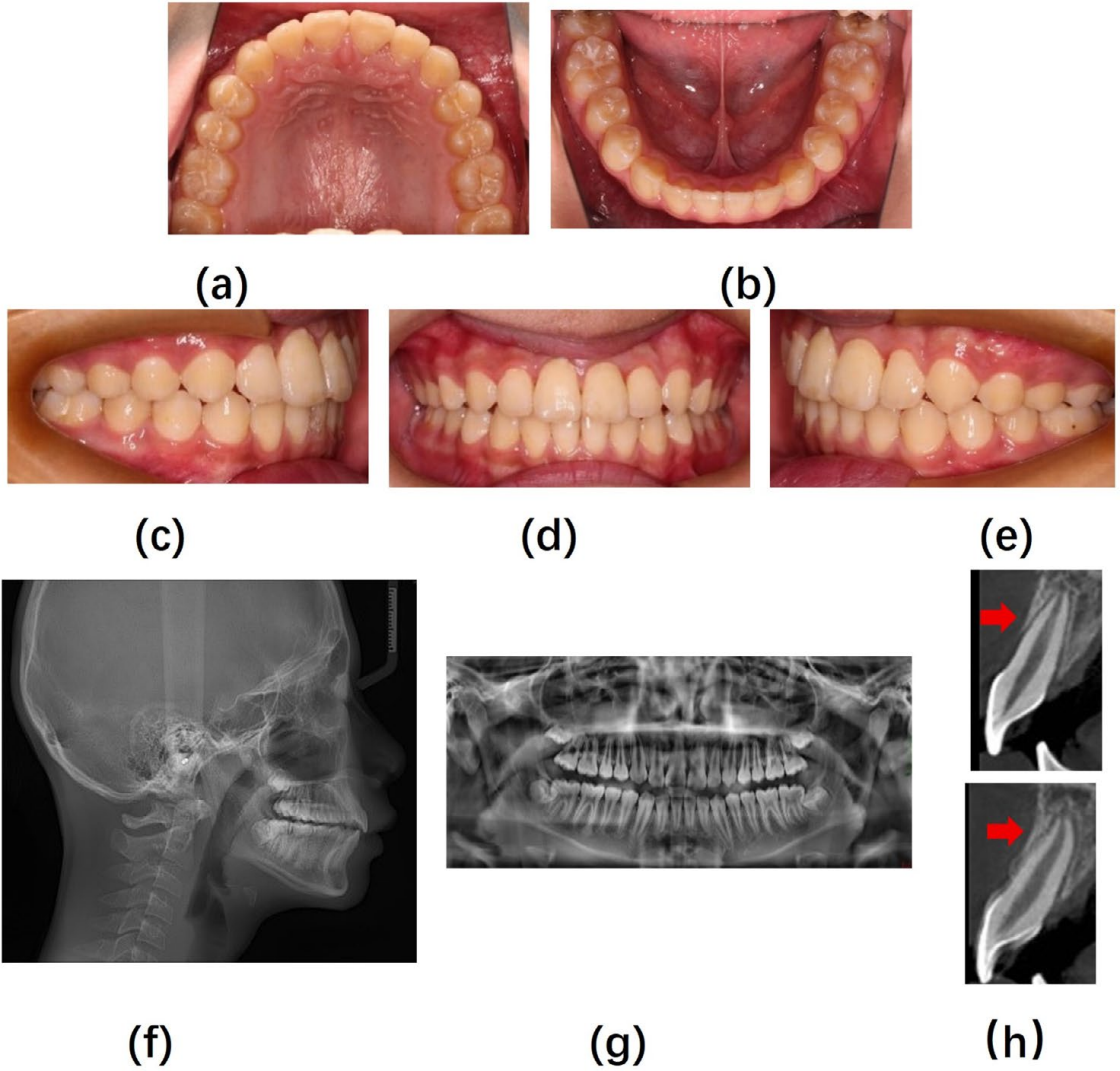
Clinical and radiograph view after orthodontic treatment. (a–e) Intraoral views after orthodontic treatment; (f–h) radiograph views after orthodontic treatment.

**FIGURE 7:**
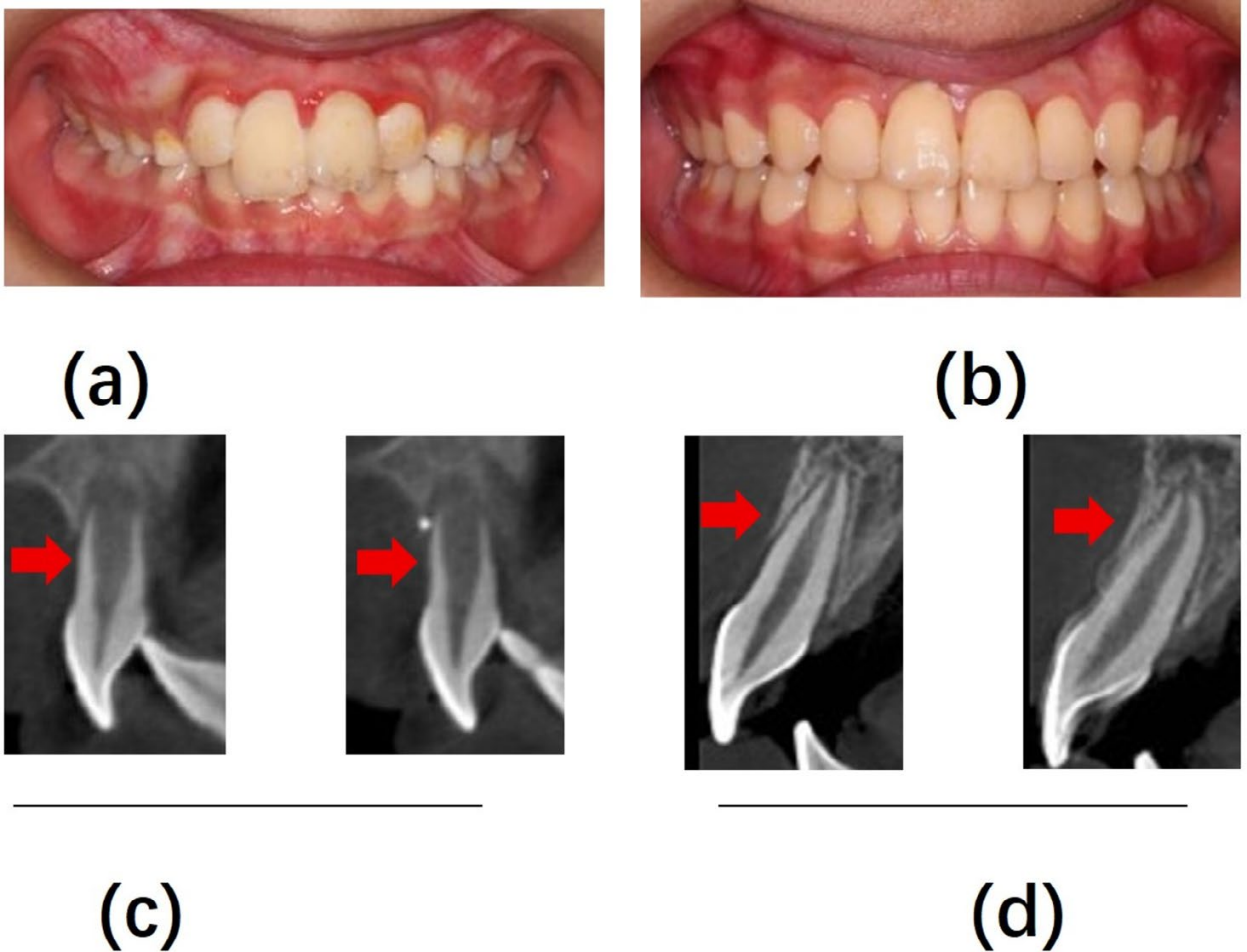
Clinical and radiograph view before and after orthodontic treatment. (a, b) Intraoral views before and after orthodontic treatment; (c–d) radiograph views before and after orthodontic treatment.

At the end of treatment, Class I molar relationship was achieved, and the gingival contours of the maxillary incisors were harmonious with the adjacent teeth. For #11 and #21, SBI was 0, CAL measured 1–2 mm, and PD was 1 mm. Post‐treatment radiographs revealed substantial alveolar bone regeneration, improved root angulation, and stable tooth positioning (Figures 6 and 7).

## 6 Discussion

This case illustrates that severe periodontal destruction resulting from misplaced orthodontic elastic bands can be effectively addressed through a prompt multidisciplinary team (MDT) strategy. Despite extensive vertical and horizontal alveolar bone loss, the treatment achieved complete resolution of inflammation, facilitated periodontal regeneration and reattachment, and maintained sustained pulp vitality without requiring endodontic intervention. Achieving timely and precise diagnosis is important for ensuring optimal clinical outcomes. Detecting periodontal damage caused by elastic bands frequently presents a diagnostic dilemma, primarily owing to the radiolucent properties of these materials and the frequent absence of explicit trauma histories among patients [5]. The pathogenic mechanism observed in this instance points to a substantial iatrogenic complication arising from previous, unaddressed orthodontic procedures. We postulate that the inciting event involved the subgingival migration of an elastic band. When an orthodontic elastic band becomes displaced into the gingival sulcus, it serves as a constant source of irritation. This condition is often responsible for the rapid onset of attachment loss and alveolar bone destruction observed in such cases.

The primary essential involves the immediate surgical extraction of the foreign irritant. Flap surgery represented the gold standard for gaining access to and decontaminating the root surfaces of compromised incisors, an approach substantiated by numerous successful case series [3, 4, 6, 7, 8]. Furthermore, comprehensive peri‐operative periodontal care is indispensable. Given that inflammation triggered by elastic forces can arise independently of plaque accumulation [9], aggressive supra‐gingival scaling is crucial to mitigate initial gingival hyper‐reactivity. Finally, post‐surgical stabilization via splint fixation constitutes a vital procedural step. To enhance healing, splinting is commonly employed to stabilize and reposition injured teeth [10]. For this case, we selected two 0.16 mm stainless steel wires for fixation. This choice aligns with recommendations for wires smaller than 0.4 mm, which offer sufficient stability while maintaining physiological mobility, particularly crucial given the open apical foramina and growth potential of the patient's immature permanent teeth [11]. Our three‐week fixation period was aligned with the 2020 IADT guidelines for dental displacement. Adhering to the 2020 Chinese Multidisciplinary Expert Consensus, we delayed active movement for 1 year to ensure periodontal stability (defined as BOP < 25%, PD < 3 mm, and mobility < Grade I). Specifically for this patient, whose oral hygiene was initially poor, treatment was postponed for 1 year to allow for clinical improvement.

To preserve periodontal health in patients with inflammation, orthodontic forces must be light and intermittent. Clear aligners are advantageous for this case; unlike fixed appliances, their thermoplastic nature generates forces that are gentle and decay rapidly after an initial peak [12, 13, 14], thereby preventing acute tissue injury from overload. Furthermore, they allow for precise regulation of both the specific teeth moved and the magnitude of their displacement [15]. The favorable outcome of this case highlights the necessity for precise biomechanical regulation during orthodontic intervention in dentition with severely diminished periodontal support. The primary clinical obstacle involved achieving intrusion of pathologically extruded incisors while avoiding exacerbation of periodontal destruction or additional alveolar bone loss. In the current protocol, clear aligners were employed to administer light, intermittent forces, restricting displacement to 0.125 mm per stage—roughly 50% of the standard rate. This approach maintains force magnitudes beneath the capillary blood pressure threshold within the periodontal ligament (PDL), thereby facilitating physiological tissue remodeling instead of inducing undermining resorption. Clear aligners acted as a removable splint by covering the entire dentition, which stabilized mobile teeth during active movement. While biofilm accumulation on aligners is debated [16], the ability to remove them was critical for this patient, given their history of poor oral hygiene. This choice aligns with meta‐analyses showing better periodontal outcomes with removable appliances [17]. Given the severity of the anterior periodontal injury and the patient's hygiene status, clear aligners were the optimal choice to ensure safe tooth movement and periodontal maintenance. Given the patient's compromised oral hygiene and the extent of periodontal damage to the anterior teeth, we prioritized an appliance that facilitates periodontal care.

## 7 Summary and Conclusions

This case underscores the importance of the cautious use of orthodontic elastic bands and the critical need for professional supervision. Effective management of iatrogenic injuries begins with proactive prevention strategies. It is imperative for clinicians to provide comprehensive instructions regarding the proper handling of orthodontic elastics. Patients must be advised to verify the seating of the elastic bands immediately after application. Furthermore, they should be warned that any signs of gingival inflammation—such as erythema, edema, or pain—or unexpected tooth mobility require immediate professional attention to prevent irreversible damage. The successful salvage of these immature teeth highlights the efficacy of a timely multidisciplinary intervention in managing complex iatrogenic periodontal trauma.

## Author Contributions

**Limin Liu:** formal analysis, writing – original draft. **Lin Chen:** methodology, writing – review and editing. **Haijing Gu:** methodology, supervision. **Xiayi Wu:** methodology. **Jieyi Chen:** writing – review and editing. **Yongbiao Huo:** formal analysis, methodology, supervision, writing – review and editing.

## Funding

The authors have nothing to report.

## Data Availability

The data supporting the findings of this study are available from the corresponding author upon reasonable request. Due to privacy or ethical restrictions, these data are not publicly available.
